# Nonesterified fatty acids during the dry period and their association with peripartum disorders, culling, and pregnancy in dairy cows

**DOI:** 10.3168/jdsc.2025-0784

**Published:** 2025-07-16

**Authors:** J. Denis-Robichaud, I. Nicola, H. Chupin, J.-P. Roy, S. Buczinski, V. Fauteux, N. Picard-Hagen, J. Dubuc

**Affiliations:** 1 Independent researcher, Amqui, Canada, G5J 2N5; 2 Faculté de médecine vétérinaire, Université de Montréal, Saint-Hyacinthe, Canada, J2S 2M2; 3 Research Centre in Food Toxicology, Université de Toulouse, Toulouse, France, 31076

## Abstract

The objective of this ambidirectional observational cohort study was to explore how nonesterified fatty acids (NEFA) 22 to 35 d before calving were related to NEFA 1 to 14 d before calving and to determine a threshold that could be used to identify cows at risk of poor postpartum health. We enrolled 855 dairy cows from 46 herds, 362 prospectively and 493 retrospectively. The NEFA concentrations were measured during the far-off period (foNEFA; 3 to 5 wk before calving) and in the close-up period (cuNEFA; up to 2 wk before calving), and postpartum infectious and metabolic disorders, reproduction success, and culling were recorded. Using a split dataset, we (1) determined a threshold maximizing the sum of sensitivity and specificity to identify peripartum conditions by classifying elevated NEFA and (2) assessed the associations between elevated NEFA and altered health and reproduction. The associations were expressed as the odds ratio (OR) and the 95% Bayesian credible interval (BCI). The concentration of foNEFA varied from 60 to 700 µmol/L (median = 149), and a threshold of ≥160 µmol/L was identified. Cows with elevated foNEFA had greater odds to have elevated cuNEFA (OR = 183, 95% BCI = 52.1–458), hyperketonemia (OR = 2.0, 95% BCI = 1.0–3.6), displaced abomasum (OR = 12.3, 95% BCI = 1.6–45.8), metritis (OR = 9.4, 95% BCI = 1.3–36.0), and clinical mastitis (OR = 5.8, 95% BCI = 1.9–12.1) than cows below the threshold. Our results suggest that foNEFA, using a threshold of ≥160 µmol/L, could be used by veterinarians as a monitoring or investigating tool to assess cows' negative energy balance before calving, even earlier than 2 wk prepartum. This monitoring could be used to implement early corrective actions to prevent the effect of negative energy balance on reproduction and peripartum health.

## Highlights

• Elevated NEFA are associated with dairy cows' altered health.
• Measured 22 to 35 days before calving, a threshold of ≥160 μmol/L can be used.
• Cows over this threshold had greater odds of metabolic and infectious diseases.
• NEFA during the dry period are early indicators of metabolic and infectious diseases.

## Graphical Abstract

**Figure:**
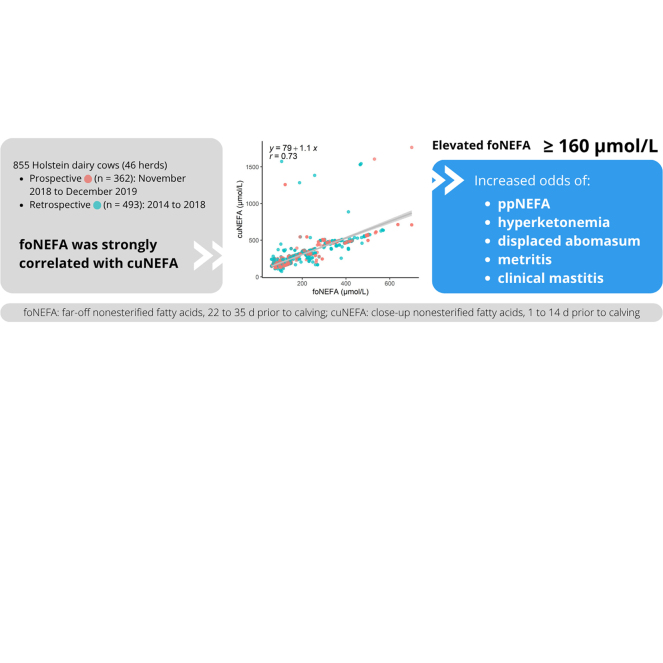
**Summary:** This study examined how nonesterified fatty acids (NEFA) in dairy cows 3 to 5 weeks before calving relate to their health and reproduction. The results showed that cows with NEFA levels at or above 160 μmol/L in this period were more likely to have elevated NEFA just before calving, along with metabolic and infectious health issues. This suggests that measuring NEFA during this far-off dry period could help veterinarians identify cows at risk of health problems during the transition period. By monitoring NEFA levels, farmers and veterinarians may better monitor and manage cows' energy balance, improving overall herd health and productivity.

The peripartum period is associated with an important increase in energy demand for dairy cows that leads to a state of negative energy balance (**NEB**). To compensate for this imbalance, cows mobilize adipose tissue, thereby releasing nonesterified fatty acids (**NEFA**), which can be converted to energy. Circulating NEFA are normal during a state of NEB, but elevated concentrations in the prepartum period have been associated with increased odds of postpartum disorders, and decreased milk production and reproductive performance (LeBlanc, 2010; Ospina et al., 2010a,b; Chapinal et al., 2011). When NEB becomes most pronounced after calving due to the high energy demands of milk production, the metabolic adjustments that precede the beginning of lactation have a critical effect in determining how effectively the cow transitions. Elevated concentration of NEFA in the close-up period (**cuNEFA**; 2 wk prepartum), defined as NEFA ≥270 or 280 µmol/L, have been shown to be associated with increased odds of postpartum disorders and decreased milk production and reproductive performance (Ospina et al., 2010a,b; Nicola et al., 2022). At the herd level, the proportion of cows with elevated cuNEFA has also been associated with poorer health, reproduction, and production outcomes (Ospina et al., 2010c; Kerwin et al., 2023; Denis-Robichaud et al., 2024).

In a veterinary clinical context, assessing cuNEFA has become a tool for monitoring peripartum NEB and health, enabling timely intervention when needed. Although implementing such a strategy during the 2 wk before calving is useful for veterinarians, nutritionists, and farmers, one could hypothesize that implementing it earlier in the dry period could allow for an earlier identification and treatment of at-risk animals, and could give information about the timing of excessive NEB (far-off vs. close-up). Unfortunately, few data are available to test this hypothesis. The main objective of this study was to explore how NEFA concentration in the far-off period (**foNEFA**) are related to cuNEFA and to identify a threshold for foNEFA at the cow level that is associated with poor postpartum health and reproduction. We also had an exploratory objective to assess the herd-level association between the proportion of cows with elevated foNEFA and cow health and reproduction.

For this ambidirectional observational cohort study, a sample size of 839 was calculated to be able to detect a 7% difference in the proportion of cows with a postpartum condition (e.g., metritis) between cows with a low or an elevated concentration of foNEFA (10% vs. 17%), considering that the ratio of cows with low and elevated foNEFA would be 3:2, with a confidence of 0.95 and a power of 0.80 (Epitools Epidemiological Calculators; Sergeant, 2018). We used the data from 855 cows (46 herds) that was obtained from 2 data sources. First, due to financial and sampling restrictions, data were collected prospectively from only 362 cows (34 herds) between November 2018 and December 2019, during a previously described project (Nicola et al., 2022). These herds were a convenience sample of regular clients of the Bovine Ambulatory Clinic of the Faculté de médecine vétérinaire of the Université de Montréal (St-Hyacinthe, QC, Canada). Criteria for including the herds were that they were located within 1 h of St-Hyacinthe (QC, Canada); that they were enrolled in a regular DHIA program, with herd health veterinary visits every 2 wk; that the research team was allowed to collect blood samples from the cows; and that the standardized disorder monitoring data were recorded in each farm's electronic health management software program. Second, we extracted a retrospective dataset (2014–2018) from 493 cows (40 herds, 28 of which were also enrolled in the prospective study), which also recorded foNEFA and collected systematic disorder monitoring data in an electronic health management software program before the study.

For the prospective data, the animal health technician collected a blood sample from all cows 42 d or less before their estimated calving date during the bi-weekly herd health visits. The samples were taken from the coccygeal vessels in tubes without anticoagulant (Vacutainer, Becton Dickinson and Company, Franklin Lakes, NJ). The tube was then kept on ice for up to 4 h and then centrifuged at 1,750 × *g* for 10 min at room temperature. The serum was harvested and stored at −20°C and sent to the Centre de diagnostic vétérinaire of the Université de Montréal (St-Hyacinthe, QC, Canada). The quantification of NEFA concentration was done on thawed samples using a Beckman DxC 600 analyzer (Beckman Coulter Corp.) and reagents supplied by Randox Laboratories Ltd. Samples analyzed for the foNEFA were the ones collected 22 to 35 d before calving. Samples for cuNEFA, collected 1 to 14 d before calving, were considered to be elevated if cuNEFA ≥280 µmol/L (Nicola et al., 2022). Farmers were blinded to the NEFA results. For the retrospective data, the blood samples were collected, analyzed, and recorded by the herd veterinarians with the same approach. Only cows with NEFA within the same timeframes (22 to 35 d and 1 to 14 d before calving) were selected.

Postpartum disorders were recorded by the farmers in a computerized recording system following standardized definitions. Retained placenta (**RP**) was defined as the lack of fetal membrane detachment 24 h after calving (Eiler and Fecteau, 2007). Metritis was defined as fetid red-brown vaginal discharge, hyperthermia (≥39.5°C), and anorexia in the first 20 d postpartum (Sheldon et al., 2006). Mastitis in the first 30 DIM was defined as the modification of the milk aspect (mild), combined with swelling of the udder (moderate), and systemic symptoms such as hyperthermia or anorexia (severe; Ruegg and Erskine, 2020). Postpartum IMI was defined as SCC ≥ 200,000 cells/mL at the first DHIA test after calving (Lactanet; Ste-Anne-de Bellevue, QC, Canada). For the different disorders, definitions used by the veterinarians of the Bovine Ambulatory Clinic were used for the prospective and retrospective data. During their herd health visits, herd veterinarians diagnosed hyperketonemia, purulent vaginal discharge (**PVD**), and endometritis. Hyperketonemia was defined as a blood sample concentration ≥1.4 mmol/L using the Precision Xtra cow-side device, between 1 and 14 DIM (Iwersen et al., 2009). Between 30 and 43 DIM, vaginal discharge was assessed with a Metricheck device (Simcro Tech Ltd., Hamilton, New Zealand) and scored as 0 (no discharge), 1 (clear mucus), 2 (flecks of purulent material), 3 (less than 50% of purulent material), 4 (50% or more of purulent material), or 5 (fetid red-brown watery discharge; McDougall et al., 2007). A score ≥3 was defined as PVD (Denis-Robichaud and Dubuc, 2015). The modified cytobrush technique was then used to collect a uterine sample (Kasimanickam et al., 2005). The cytobrush was placed in 1 mL of saline and shaken. To quantify the amount of leukocytes and diagnose endometritis, a commercial strip (Multistix 10 SG; Bayer Corporation, Elkhart, IN) was placed in the solution and scored after 2 min as 0 (negative), 0.5 (traces of leukocytes), 1 (small amount of leukocytes), 2 (moderate amount of leukocytes), or 3 (large amount of leukocytes). Endometritis was defined as a score ≥1 (Denis-Robichaud and Dubuc, 2015). Pregnancy following inseminations was assessed and recorded by the herd veterinarian during regular visits. Displaced abomasum (**DA**) was diagnosed by veterinarians, when digestive signs were present, as a distinctive pinging noise during the auscultation and percussion of the left or right flank.

We conducted the analyses with R (version 4.3.0) using the R Studio interface (version 2021.09.0; R Core Team, 2015). For cow-level variables, parity was categorized as first, second, or third and greater, according to the lactation preceding the dry period during which cows were enrolled. Season of calving was categorized as winter (January to March), spring (April to June), summer (July to September), or fall (October to December). For herd-level variables, herd size was recorded as a continuous variable, feeding systems were categorized as TMR or conventional, milking systems were categorized as robot, parlor or pipeline, and housing was categorized as tiestall or freestall. After obtaining descriptive analyses, we assessed the correlation between foNEFA and cuNEFA. We considered a correlation coefficient (Pearson: r, Spearman: ρ) of 0 to ± 0.39 weak, of ± 0.40 to ± 0.69 moderate, and of > ± 0.70 strong (Dancey and Reidy, 2007). The dataset was then split randomly into a training dataset and a test dataset (60:40 ratio). Using the training dataset, we calculated for each disorder the area under the curve (**AUC**) of the receiver operating characteristic curve for foNEFA (cutpointr package; Thiele and Hirschfeld, 2021). An AUC <0.7 was considered poor, an AUC of 0.7 to <0.8 was considered acceptable, an AUC of 0.8 to <0.9 was considered excellent, and an AUC ≥0.9 was considered outstanding (Mandrekar, 2010; Hosmer et al., 2013). We then identified, for each disorder, the threshold that would maximize the sum of sensitivity and specificity. We calculated the 95% CI for the AUC, sensitivity, and specificity using the bootstrapping approach. As multiple thresholds were identified, we selected the median threshold for the cow- and herd-level analyses. Using the test dataset, we explored the association between foNEFA (dichotomized with the median threshold) and multiple outcomes. For these models, cows were the experimental unit, and one Bayesian logistic regression model per outcome (cuNEFA, postpartum disorders, reproductive success, and culling before 100 DIM) was built (brms package). For all models, the exposure of interest was foNEFA, parity, season, and study design (prospective or retrospective) were included as confounders, and aggregation by herd was adjusted with random intercepts.

For the herd-level associations, we used only the prospective study data to ensure that the selection of cows was systematic, and we included herds for which we had at least 12 cows enrolled. For these models, herds were the experimental unit. To evaluate the associations between the proportion of cows with elevated foNEFA (as a continuous variable) and the proportion of each outcome (elevated cuNEFA, postpartum disorders, success at first insemination, and culling before 100 DIM), we used Bayesian aggregated binomial regression models. Models were adjusted for the feeding and milking systems used on the farm, and the herd size as confounders. As housing and milking systems were colinear (all tiestall barns had pipeline milking systems), housing was not included as a confounder.

We used weakly informative priors in all models. For the logistic regression models, we used flat priors for the regression coefficients and Student-t (df = 3, µ = 0, σ = 2.5) priors for the intercept and the standard deviations (McElreath, 2020). For the aggregated binomial regression models, we used normal (µ = 0, σ^2^ = 1) priors for the intercept and the regression coefficients. For all models, we used 3 chains with a length of 3,000, in which the first 1,000 iterations were used as a warm-up to generate the models (Homan and Gelman, 2014). We monitored convergence by visually inspecting the trace plots of variance components and density plots and by ensuring that we obtained an effective sample size of 1,000 or greater (Bürkner, 2017). The odds ratio (**OR**), or the OR for every 10-point increase in the proportion of cows with elevated foNEFA (**OR_10%_**), marginal means, and 95% Bayesian credible intervals (**BCI**) were calculated.

For cow-level analyses, we had data from 855 Holstein cows in 46 herds (1–93 cows per herd), with 0 to 376 missing values, depending on the disorder (Table 1). Only 13 cows had data for 2 lactations. Cows were in the dry period following their first (n = 157; 19%), second (n = 268; 31%), or third and greater (n = 430; 50%) lactation. Calvings were in spring (n = 298; 35%), summer (n = 120; 14%), fall (n = 177; 21%), and winter (n = 260; 30%). The proportion of cows with the different peripartum disorders varied from 5% for culling before 100 DIM to 49% for cuNEFA (Table 1). The proportion of success at first artificial insemination (**AI**) was 40% (n = 322/807). The foNEFA concentration varied from 60 to 700 µmol/L (median = 149) and were strongly correlated with the cuNEFA concentration (r = 0.73, ρ = 0.85; Figure 1). The distribution of foNEFA for cows with and without different conditions suggested that this could identify cows at risk of postpartum health issues (Table 1). However, the AUC values for foNEFA were poor for almost all conditions, but the AUC for DA was acceptable and for cuNEFA was outstanding (Table 1). The thresholds maximizing the sum of sensitivity and specificity varied between ≥128 and 192 µmol/L for foNEFA (median = 162). Using a threshold of ≥160 µmol/L, we found that cows with elevated foNEFA had greater odds to have elevated cuNEFA, hyperketonemia, DA, metritis, and clinical mastitis than cows below the threshold (Table 2).

**Table 1:** Proportions of cows with each peripartum disorder, and concentrations of nonesterified fatty acids (NEFA) 22 to 35 d before calving (foNEFA) for cows with and without each condition

Disorder^3^	Proportion, % (n/n)	Median foNEFA, µmol/L (IQR^1^)		Predicted condition^2^			
		Disorder present	Disorder absent	AUC (95% CI)	Threshold	Se (95% CI)	Sp (95% CI)
cuNEFA	49 (420/855)	232 (181–355)	108 (92–129)	0.96 (0.94–0.97)	≥152	0.92 (0.90–0.95)	0.93 (0.90–0.96)
Hyperketonemia	21 (172/836)	175 (110–246)	145 (104–232)	0.56 (0.51–0.61)	≥161	0.60 (0.46–0.72)	0.58 (0.45–0.70)
DA	8 (66/840)	238 (179–426)	144 (105–223)	0.71 (0.64–0.78)	≥192	0.69 (0.56–0.84)	0.68 (0.58–0.79)
RP	9 (77/855)	193 (127–298)	147 (106–232)	0.63 (0.56–0.69)	≥163	0.74 (0.58–0.95)	0.56 (0.30–0.67)
Metritis	18 (153/840)	193 (136–298)	140 (104–223)	0.66 (0.62–0.71)	≥170	0.72 (0.62–0.82)	0.62 (0.51–0.68)
PVD	12 (98/840)	146 (105–234)	149 (107–234)	0.51 (0.44–0.57)	≥162	0.47 (0.11–0.53)	0.68 (0.56–0.95)
Endometritis	35 (170/479)	146 (100–237)	149 (110–234)	0.53 (0.47–0.59)	≥128	0.72 (0.45–0.87)	0.42 (0.23–0.68)
IMI	25 (211/829)	172 (116–230)	141 (104–237)	0.57 (0.52–0.61)	≥147	0.70 (0.61–0.78)	0.53 (0.47–0.61)
CM100	22 (181/840)	192 (139–298)	138 (103–223)	0.66 (0.61–0.71)	≥163	0.74 (0.65–0.81)	0.59 (0.53–0.65)
CUL100	5 (44/855)	158 (106–211)	149 (107–211)	0.50 (0.41–0.60)	≥150	0.63 (0.43–0.88)	0.55 (0.20–0.65)

1 IQR = interquartile range.

2 Additionally, the table shows, from the training dataset (n = 513), the area under the curve (AUC) for predicting each condition with foNEFA and the foNEFA threshold (µmol/L) maximizing the sum of sensitivity (Se) and specificity (Sp) to discriminate each condition.

3 cuNEFA = elevated NEFA in the close-up period (≥280 µmol/L; 1 to 14 d before calving); IMI = SCC ≥200,000 at the first DHIA test (5–71 DIM); CM100 = clinical mastitis before 100 DIM; CUL100 = culled before 100 DIM.

**Figure 1:**
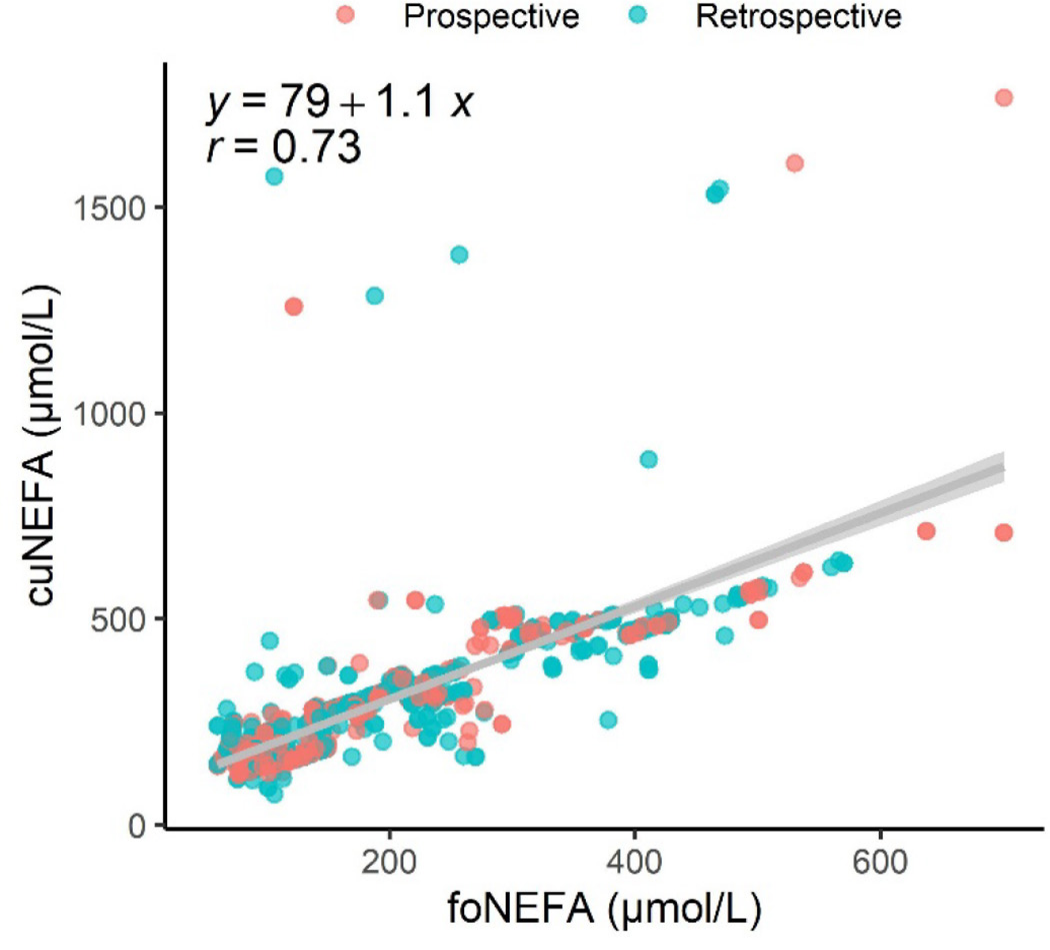
Scatter plot and linear association (95% CI; gray shaded area) of NEFA in the far-off period (foNEFA; 22 to 35 d before calving) and the close-up period (cuNEFA; 1 to 14 d before calving). Samples were collected from a prospective phase (n = 362) and a retrospective phase (n = 493).

**Table 2:** Estimated proportions of cows with each disorder, according to their foNEFA status, and OR of having each condition when foNEFA were elevated (n = 342; test dataset)^1^

Disorder^3^	Estimated proportion (95% BCI)		OR^2^ (95% BCI)	
	foNEFA <160	foNEFA ≥160	Unadjusted	Adjusted
cuNEFA	0.09 (0.04–0.15)	0.94 (0.90–0.98)	**127 (42.9–281)**	**183 (52.1–458)**
Hyperketonemia	0.12 (0.06–0.18)	0.21 (0.13–0.30)	1.76 (0.84–2.93)	**2.03 (1.01–3.60)**
DA	0.01 (0.00–0.02)	0.05 (0.01–0.10)	**6.89 (1.38–20.3)**	**12.3 (1.57–45.8)**
RP	0.06 (0.03–0.11)	0.12 (0.06–0.18)	2.01 (0.90–3.83)	2.05 (0.80.93–3)
Metritis	0.01 (0.00–0.02)	0.04 (0.01–0.14)	**2.14 (1.05–3.91)**	**9.39 (1.26–36.0)**
PVD	0.04 (0.00–0.10)	0.03 (0.00–0.07)	0.61 (0.22–1.25)	0.72 (0.22–1.61)
Endometritis	0.05 (0.00–0.15)	0.05 (0.00–0.14)	1.20 (0.43–2.38)	0.94 (0.26–2.16)
S1AI	0.41 (0.31–0.51)	0.35 (0.26–0.46)	0.88 (0.51–1.35)	0.80 (0.46–1.27)
IMI	0.20 (0.11–0.31)	0.28 (0.17–0.39)	1.42 (0.78–2.24)	1.49 (0.69–2.50)
CM100	0.05 (0.01–0.11)	0.25 (0.10–0.41)	**3.40 (1.56–5.90)**	**5.78 (1.88–12.1)**
CUL100	0.03 (0.01–0.06)	0.02 (0.00–0.05)	0.66 (0.12–1.55)	0.71 (0.15–1.84)

1 All models addressed aggregation by herd (random intercept), and adjusted models included for parity and season, and whether the data were from the prospective or the retrospective study design as fixed effects.

2 The reference is cows with foNEFA <160 µmol/L (if bold, the 95% CI does not include 1).

3 cuNEFA = elevated NEFA in the close-up period (≥280 µmol/L; 1 to 14 d before calving); S1AI = success at first insemination; IMI = SCC ≥200,000 at first DHIA test; CM100 = clinical mastitis before 100 DIM; CUL100 = culled before 100 DIM.

At the herd level, 12 herds were included in the analyses (n = 269 cows; 12–58 cows per herd) as 12 herds had only retrospective data, and 22 herds had fewer than 12 observations (1–11, median = 4). The herd-level prevalence of cows with elevated foNEFA was between 14% and 71% (median = 47%). For every 10-point increase in the proportion of cows with elevated foNEFA, the odds of cuNEFA in the herd increased (OR_10%_ = 1.88, 95% BCI = 1.25–2.86), as did the odds of metritis (OR_10%_ = 4.88, 95% BCI = 2.76–9.32), mastitis (OR_10%_ = 3.32, 95% CI = 1.97–5.88), and IMI (OR_10%_ = 2.09, 95% BCI = 1.36–3.38). The odds of success at first insemination decreased (OR_10%_ = 0.65, 95% BCI = 0.43–0.97) for every 10-point increase in the proportion of cows with elevated foNEFA. The odds of hyperketonemia in the herd also decreased for every 10-point increase in proportion of cows with elevated foNEFA (OR_10%_ = 0.52, 95% BCI = 0.32–0.82).

This study allowed us to identify a strong correlation between foNEFA and cuNEFA, suggesting that NEFA could give information on cows' NEB and health 3 to 5 wk before calving. The thresholds we identified, and the one we selected (≥160 µmol/L), in the 22 to 35 d before calving were lower than those identified 1 to 14 d before calving (≥270 to 300 µmol/L; Ospina et al., 2010b; Chapinal et al., 2011; Nicola et al., 2022). With our selected threshold, elevated foNEFA were associated not only with elevated cuNEFA but also with increased odds of postpartum hyperketonemia, DA, metritis, and mastitis. Elevated cuNEFA were also associated with these conditions in other studies, but also with RP, reproduction, and culling, although often with a higher threshold (Ospina et al., 2010a,b; Chapinal et al., 2011; Roberts et al., 2012; Nicola et al., 2022). It is unclear why we did not observe these associations in our results, but the absence of associations of foNEFA with success at first insemination and with culling might be due to the limited sample size of the present study and a weak biological link between them, and to the long time between exposure and outcome, leaving time for other health events to occur and blur a potential association. Additionally, we did not have data to explore why cows with elevated foNEFA had greater odds to have hyperketonemia, but herds with higher proportion of cows with elevated foNEFA had lower odds of hyperketonemia. We could, however, hypothesize that individual cows with elevated foNEFA have long-term effecs and greater odds of hyperketonemia, but herds with a high proportion of cows with elevated foNEFA could have a close-up ration supporting high-producing cows and manage the herd well enough to prevent hyperketonemia, leading to lower overall odds of hyperketonemia.

The retrospective subsample in the present study could have influenced our findings. Indeed, cows in this subsample were not systematically enrolled. They were most likely sampled by veterinarians monitoring or investigating peripartum issues. As no treatment is used for cows according to their foNEFA, we still believe that the cows in this subsample were not sick cows but perhaps cows from herds with transition challenges. Although this has likely affected the prevalence of elevated NEFA concentration and peripartum conditions (Denis-Robichaud et al., 2024), we think that the threshold and the association between foNEFA and disorders were minimally impacted. The conservative interpretation of our results, however, is that the threshold and associations are for herds with transition challenges. A similar study could be repeated in different contexts to strengthen our findings.

Although we think that the selection bias we faced had a limited effect on our main objective, it had more potential to affect our exploratory objective. Indeed, the NEFA concentration and disorder prevalence in herds with cows from the retrospective subsample are likely inadequate to assess the association between foNEFA and peripartum conditions at the herd level, and we kept only the samples from cows that were selected prospectively. Consequently, the sample for this objective was limited and should be interpreted with caution, but we still found herd-level associations between foNEFA and peripartum conditions and success at first insemination. These associations are similar to the ones we found between cuNEFA concentration and metritis and success at first AI (Denis-Robichaud et al., 2024). The additional associations in the present study are with IMI and mastitis. As for metritis, the increased odds of IMI and mastitis could be due to elevated NEFA affecting immune functions (Lacasse et al., 2018), but we cannot exclude that a bias, for example a selection or a confounding bias, could have affected our results given the small sample size for this exploratory question.

Nonetheless, our results highlight a strong association between foNEFA and cuNEFA, as well as increased odds of postpartum conditions for cows with elevated foNEFA. Moreover, the herd prevalence of elevated foNEFA was associated with the odds of peripartum conditions and success at first AI, although these results need to be interpreted with caution due to the study design. Our findings can be useful to veterinarians who want to monitor or investigate NEB problems during the dry period. Indeed, cuNEFA concentration have been used to identify cows at risk of altered health, reproduction, and production earlier than what can be done with other tools such as postpartum BHB (Ospina et al., 2010a,b; Chapinal et al., 2011; Roberts et al., 2012), and our findings highlight that there is a strong correlation between foNEFA and cuNEFA. This suggests that it might be possible to identify NEB before calving earlier and that interventions on housing or feeding strategies during the dry period can perhaps be implemented earlier to minimize the effect on postpartum health and reproduction. The latter remains to be investigated. The strong association between foNEFA and cuNEFA also suggests that testing 3 to 5 wk before calving is likely to give the same information as testing in 2 wk before calving. This could hinder the ability to discriminate between an excessive NEB due to late lactation or early dry issues and an excessive NEB due to transition problems.
